# Serum midkine levels are increased in patients with various types of carcinomas

**DOI:** 10.1054/bjoc.2000.1339

**Published:** 2000-08-17

**Authors:** S Ikematsu, A Yano, K Aridome, M Kikuchi, H Kumai, H Nagano, K Okamoto, M Oda, S Sakuma, T Aikou, H Muramatsu, K Kadomatsu, T Muramatsu

**Affiliations:** Meiji Cell Technology Center, 540 Naruda, Odawara, 250–0862; Department of Surgery, Faculty of Medicine, Kagoshima University, 8–35–1 Sakuragaoka, Kagoshima, 890; Departments of Medical Technology, Surgery, University of Occupational and Environmental Health, 1–1 Iseigaoka, Yahatanishi-ku, Kitakyushu, 807–0804; Department of Biochemistry, Nagoya University School of Medicine, 65 Tsurumai-cho, Showa-ku, Nagoya, 466–8550, Japan

**Keywords:** enzyme-linked immunoassay, growth factor, midkine, serum, tumour marker

## Abstract

The level of expression of midkine (MK), a heparin-binding growth factor, is increased in many types of human carcinomas. An enzyme-linked immunoassay, which utilizes a combination of rabbit and chicken antibodies revealed that serum MK level in the controls (*n*= 135) was 0.154 ± 0.076 (mean ± SD) ng ml^–1^with an apparent cut-off value as 0.5 ng ml^–1^. Serum MK level was significantly elevated in the cancer patients (*n*= 150) (*P*< 0.001); 87% of the patients showed levels of more than 0.5 ng ml^–1^. All ten types of cancer examined showed a similar profile of serum MK level. There was no or weak correlation between C-reactive protein level, a marker of inflammation, and serum MK level. Furthermore, in case of gastric carcinoma and lung carcinoma, patients with stage I carcinoma already showed elevated serum MK levels. The present results indicated that serum MK could serve as a general tumour marker with a good potential for clinical application. © 2000 Cancer Research Campaign


British Journal of Cancer (2000) 83(6), 701–706
© 2000 Cancer Research Campaign
doi: 10.1054/ bjoc.2000.1339, available online at http://www.idealibrary.com on IDEAL®

### Serum midkine levels are increased in patients with various types of carcinomas

**S Ikematsu[1], A Yano[1], K Aridome[2], M Kikuchi[3], H Kumai[1], H Nagano[1], K Okamoto[4], M Oda[1], S Sakuma[1], T Aikou[2], H Muramatsu[5], K Kadomatsu[5] and T Muramatsu[5]**

[1]Meiji Cell Technology Center, 540 Naruda, Odawara 250–0862; [2]Department of Surgery, Faculty of Medicine, Kagoshima University, 8–35–1 Sakuragaoka, Kagoshima 890; [3]Departments of Medical Technology and [4]Surgery, University of Occupational and Environmental Health, 1–1 Iseigaoka, Yahatanishi-ku, Kitakyushu 807–0804; [5]Department of Biochemistry, Nagoya University School of Medicine, 65 Tsurumai-cho, Showa-ku, Nagoya 466–8550, Japan

**Summary** The level of expression of midkine (MK), a heparin-binding growth factor, is increased in many types of human carcinomas. An enzyme-linked immunoassay, which utilizes a combination of rabbit and chicken antibodies revealed that serum MK level in the controls ($n$ = 135) was 0.154 ± 0.076 (mean ± SD) ng ml$^{-1}$ with an apparent cut-off value as 0.5 ng ml$^{-1}$. Serum MK level was significantly elevated in the cancer patients ($n$ = 150) ($P < 0.001$); 87% of the patients showed levels of more than 0.5 ng ml$^{-1}$. All ten types of cancer examined showed a similar profile of serum MK level. There was no or weak correlation between C-reactive protein level, a marker of inflammation, and serum MK level. Furthermore, in case of gastric carcinoma and lung carcinoma, patients with stage I carcinoma already showed elevated serum MK levels. The present results indicated that serum MK could serve as a general tumour marker with a good potential for clinical application.
© 2000 Cancer Research Campaign

**Keywords**: enzyme-linked immunoassay; growth factor; midkine; serum; tumour marker

Midkine (MK) is a heparin-binding growth factor found as the product of a retinoic acid-responsive gene (Kadomatsu et al, 1988). It has 45% sequence identity to pleiotrophin (PTN)/heparin-binding growth-associated molecule (HB-GAM), and together these molecules comprise a family of heparin-binding growth factors (Li et al, 1990; Merenmies and Rauvala, 1990). MK has diverse activities, for example it enhances neuronal cell survival, neurite outgrowth and fibrinolysis (Michikawa et al, 1993; Muramatsu et al, 1993; Kojima et al, 1995). MK as well as PTN/HB-GAM have been implicated in carcinogenesis (Kurtz et al, 1995). MK transforms NIH3T3 cells (Kadomatsu et al, 1997) and enhances angiogenic activities of tumour cells (Choudhuri et al, 1997). It is highly expressed at both mRNA and protein levels in premalignant stages in human colorectal carcinomas (Ye et al, 1999). MK mRNA and protein expression are frequently elevated in many human carcinomas, such as breast, lung, oesophageal, stomach, colorectal, liver, ovary and urinary bladder carcinomas, prostate carcinomas, glioblastomas, neuroblastomas and Wilms' tumours (Tsutsui et al, 1993; Garver et al, 1994; Aridome et al, 1995; Nakagawara et al, 1995; O'Brien et al, 1996; Mishima et al, 1997; Konishi et al, 1999). In neuroblastomas, glioblastomas and urinary bladder carcinomas, high levels of MK expression are correlated with poor prognosis (Nakagawara et al, 1995; O'Brien et al, 1996; Mishima et al, 1997). Since MK is a secreted protein, serum MK levels are expected to increase when tumour tissues express abundant MK. Although our preliminary studies yielded promising results, they were not conclusive because of the limited numbers of samples (Song et al, 1997). In the present study, we employed a new and more convenient assay method and analysed samples from 150 cancer patients and 135 controls.

*Received 24 November 1999*
*Revised 11 May 2000*
*Accepted 17 May 2000*

*Correspondence to:* T Muramatsu

### MATERIALS AND METHODS

### Human MK and antibodies

To generate human MK protein, an expression vector for yeast (*Pichia pastoris* GS115; Research Corporation Technologies) was constructed by inserting a cDNA fragment covering the open reading frame of human MK into pHIL-D4 (In Vitrogen). Following transfection of the expression vector into yeast, selection with histidine and G418 was carried out. The human MK protein was purified from yeast by anion exchange chromatography and affinity chromatography on a heparin column. The purified protein exhibited neurotrophic activity comparable with that of L cell-produced mouse MK (Muramatsu et al, 1993).

Rabbit and chicken antibodies were raised against the yeast-produced human MK, and were precipitated with ammonium sulphate, and affinity purified with a human MK-column. These antibodies specifically detected human MK on Western blotting analysis (data not shown). Peroxidase labelling of the antibodies was carried out as described (Ishikawa et al, 1993).

### Serum samples

Sera were obtained from blood collected from normal individuals ($n$ = 135; male = 94; female = 41; ages 20–29 = 31; 30–39 = 59; 40–49 = 25; 50–59 = 11; > 60 = 9; median value = 34; average value = 37.6), cancer patients ($n$ = 150; male = 96; female = 54; ages 26–85; median value = 64; average value = 62.9) (Table 1) and other patients with or without inflammation ($n$ = 80; male = 35; female =

**Table 1** Clinical characteristics of tumours

| | *n* |
|---|---|
| **Oesophageal carcinoma** | |
| Histology | |
| Well differentiated squamous cell carcinoma | 6 |
| Moderately differentiated squamous cell carcinoma | 5 |
| Poorly differentiated squamous cell carcinoma | 3 |
| Squamous cell carcinoma | 2 |
| Adenosquamous cell carcinoma | 2 |
| Stage | |
| I | 5 |
| II | 4 |
| III | 6 |
| IV | 2 |
| Recurrence | 1 |
| Size | |
| ≤ 2 cm | 0 |
| 2–5 cm | 10 |
| ≥ 5 cm | 8 |
| **Gastric carcinoma** | |
| Histology | |
| Well differentiated tubular adenocarcinoma | 6 |
| Well to moderately differentiated tubular adenocarcinoma | 1 |
| Moderately differentiated tubular adenocarcinoma | 5 |
| Poorly differentiated adenocarcinoma | 9 |
| Mucinous adenocarcinoma | 1 |
| Adenocarcinoma (unclassified) | 2 |
| signet-ring cell carcinoma | 6 |
| Stage | |
| I | 18 |
| II | 2 |
| III | 6 |
| IV | 4 |
| Size | |
| ≤ 2 cm | 5 |
| 2–5 cm | 12 |
| ≥ 5 cm | 11 |
| No surgery | 2 |
| **Duodenal carcinoma (adenocarcinoma)** | |
| Stage | |
| I | 0 |
| II | 0 |
| III | 2 |
| IV | 0 |
| Size | |
| ≤ 2 cm | 0 |
| 2–5 cm | 1 |
| ≥ 5 cm | 1 |
| **Colon carcinoma** | |
| Histology | |
| Well differentiated adenocarcinoma | 10 |
| Well to moderately differentiated adenocarcinoma | 1 |
| Moderately differentiated adenocarcinoma | 6 |
| Mucinous cystadenocarcinoma | 1 |
| Adenocarcinoma (unclassified) | 6 |
| Focal carcinoma in tubular adenoma | 1 |
| Stage | |
| 0 | 1 |
| I | 4 |
| II | 4 |
| III | 5 |
| IV | 5 |
| Recurrence | 6 |
| Size | |
| ≤ 2 cm | 3 |
| 2–5 cm | 10 |
| ≥ 5 cm | 11 |
| No surgery | 1 |
| **Hepatocellular carcinoma** | |
| Stage | |
| I | 0 |
| II | 11 |
| III | 9 |
| IV | 2 |
| Recurrence | 3 |
| Size | |
| ≤ 2 cm | 3 |
| 2–5 cm | 15 |
| ≥ 5 cm | 6 |
| No surgery | 1 |
| **Bile-duct and gallbladder carcinoma** | |
| Histology | |
| Well differentiated adenocarcinoma | 2 |
| Moderately differentiated adenocarcinoma | 4 |
| Poorly to moderately differentiated adenocarcinoma | 1 |
| Poorly differentiated adenocarcinoma | 1 |
| Tubular adenocarcinoma | 1 |
| Cholangiocarcinoma | 1 |
| No surgery | 2 |
| Stage | |
| I | 2 |
| II | 4 |
| III | 4 |
| IV | 2 |
| Size | |
| ≤ 2 cm | 3 |
| 2–5 cm | 5 |
| ≥ 5 cm | 2 |
| No surgery | 2 |
| **Pancreatic carcinoma** | |
| Histology | |
| Invasive ductal carcinoma | 2 |
| Adenocarcinoma | 4 |
| Papillary adenocarcinoma | 2 |
| Anaplastic ductal carcinoma | 1 |
| Stage | |
| I | 5 |
| II | 0 |
| III | 1 |
| IV | 3 |
| Size | |
| ≤ 2 cm | 2 |
| 2–5 cm | 3 |
| ≥ 5 cm | 1 |
| No surgery | 3 |
| **Thyroid carcinoma (papillary carcinoma)** | |
| Stage | |
| I | 0 |
| II | 4 |
| III | 1 |
| IV | 0 |
| Size | |
| ≤ 2 cm | 3 |
| 2–5 cm | 2 |
| ≥ 5 cm | 0 |
| **Lung carcinoma** | |
| Histology | |
| Well differentiated adenocarcinoma | 3 |
| Moderately differentiated adenocarcinoma | 9 |
| Poorly differentiated adenocarcinoma | 1 |
| Adenocarcinoma (unclassified) | 1 |
| Small cell carcinoma | 1 |
| Squamous cell carcinoma | 3 |
| Papillary adenocarcinoma with large cell carcinoma | 1 |
| Stage | |
| I | 11 |
| II | 1 |
| III | 6 |
| IV | 1 |
| Size | |
| ≤ 2 cm | 3 |
| 2–5 cm | 14 |
| ≥ 5 cm | 2 |

© 2000 Cancer Research Campaign

**Breast carcinoma**
Table 1 continued

| | *n* |
|---|---|
| Histology | |
| Invasive ductal carcinoma | 4 |
| Noninvasive ductal carcinoma | 1 |
| Stage | |
| I | 1 |
| II | 3 |
| III | 0 |
| IV | 1 |
| Size | |
| ≤ 2 cm | 1 |
| 2–5 cm | 3 |
| ≥ 5 cm (1) | 1 |

45; ages 20–70), and were immediately frozen and kept at –20°C until assay. We regard those with C-reactive protein (CRP) values > 0.1 mg ml$^{-1}$ as those with inflammation and those with CRP values < 0.1 mg ml$^{-1}$ as those without inflammation. Except otherwise specified, sera from cancer patients were obtained before surgical or other treatment. Informed consent was obtained from patients and normal individuals according to the guidelines approved by Ethical Committee of the University Hospitals. Stage of carcinoma was determined by the TNM classification. C-reactive protein level was determined by latex aggregation method.

### Enzyme-linked immunoassay (EIA) for human MK

As a standard method, 50 µl of rabbit anti-human MK antibodies at a concentration of 5.5 µg ml$^{-1}$ in phosphate-buffered saline (PBS) was adsorbed onto the wells of microtiter plates (Polysorp plates, Nunc) for 16 h at room temperature. After washing with 1% Tween 20 in PBS, the wells were blocked with 150 µl of 0.5% BSA in PBS for 2 h at 37°C. On the other hand, control human MK samples or serum samples (10 µl each) were mixed with 100 µl of 50 mM Tris HCl (pH 8.4), 0.5 M KCl, 0.5% bovine serum albumin (BSA), 0.01% Microcide I (aMReSCO, Solon, Ohio, USA) containing peroxidase-labelled chicken anti-human MK antibodies (0.1 µg ml$^{-1}$). Aliquots of 50 µl of this mixture were added into the wells of microtiter plates prepared as described above. After incubation for 1 h at room temperature, the wells were washed five times with 1% Tween 20 in PBS. Aliquots of 100 µl of a substrate solution (tetramethylbenzidine at 0.5 mg ml$^{-1}$ in Dako S1600, Dako, USA) were added into the wells and the plates were incubated for 30 min at room temperature. The reaction was stopped by adding 100 µl of 2 N sulphuric acid, and OD450 was detected using a multiplate reader (Model 3550, BioRad).

### Statistics

Statistical significance was evaluated by Mann–Whitney U test. Correlation was examined by Spearman's correlation coefficient by rank with *Z* conversion of Fisher's *r*.

### RESULTS

### EIA for MK employing two polyclonal antibodies from different species

The newly developed EIA for human MK employed a combination of two anti-human MK antibodies from different species

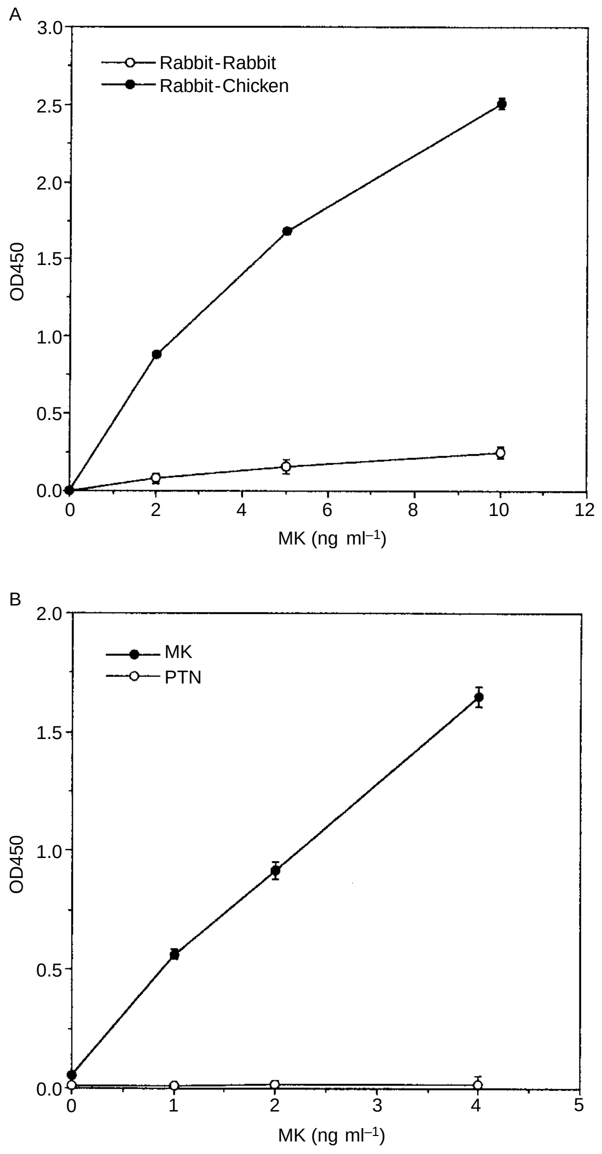

**Figure 1** Enzyme-linked immunoassay for human MK. **A** The combination of rabbit and chicken antibodies showed higher sensitivity than use of rabbit antibodies alone. Each point was the average of five assays ± SD. **B** Absence of cross-reactivity with human pleiotrophin. The EIA for human MK barely detected human pleiotrophin

(rabbit and chicken). This procedure increased the sensitivity of the detection of human MK, as compared with the use of rabbit antibodies alone (Figure 1**A**). No cross-reactivity was observed with human pleiotrophin, which shows 45% sequence identity to human MK (Figure 1**B**).

### Serum MK levels of normal controls and cancer patients

Using this EIA, we determined serum MK levels of 135 control individuals. Normal serum MK level was 0.154 ± 0.076 ng ml$^{-1}$ (mean ± SD), and in no case did the value reach 0.5 ng ml$^{-1}$ (Figure 2). Thus 0.5 ng ml$^{-1}$ was set as the cut-off value. There were no significant differences in MK values between men and

© 2000 Cancer Research Campaign 

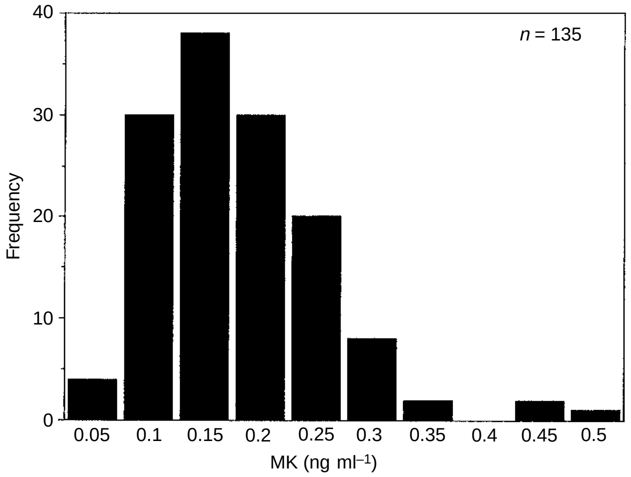

**Figure 2** Serum MK level of normal controls. Each number on the x-axis indicates the upper value of serum MK level in increments of 0.05 ng ml$^{-1}$

**Table 2** Serum MK levels in patients with gastric carcinomas and lung carcinomas at different stages

| | Serum MK (ng ml$^{-1}$) Stage I | II–IV |
|---|---|---|
| Gastric carcinoma ($n$ = 31) | 0.73 ($n$ = 18) | 0.93 ($n$ = 13) |
| Lung carcinoma ($n$ = 21) | 1.21 ($n$ = 11) | 2.05 ($n$ = 8) |

All values larger than the average normal serum value ($P$ < 0.01, Mann–Whitney U test)

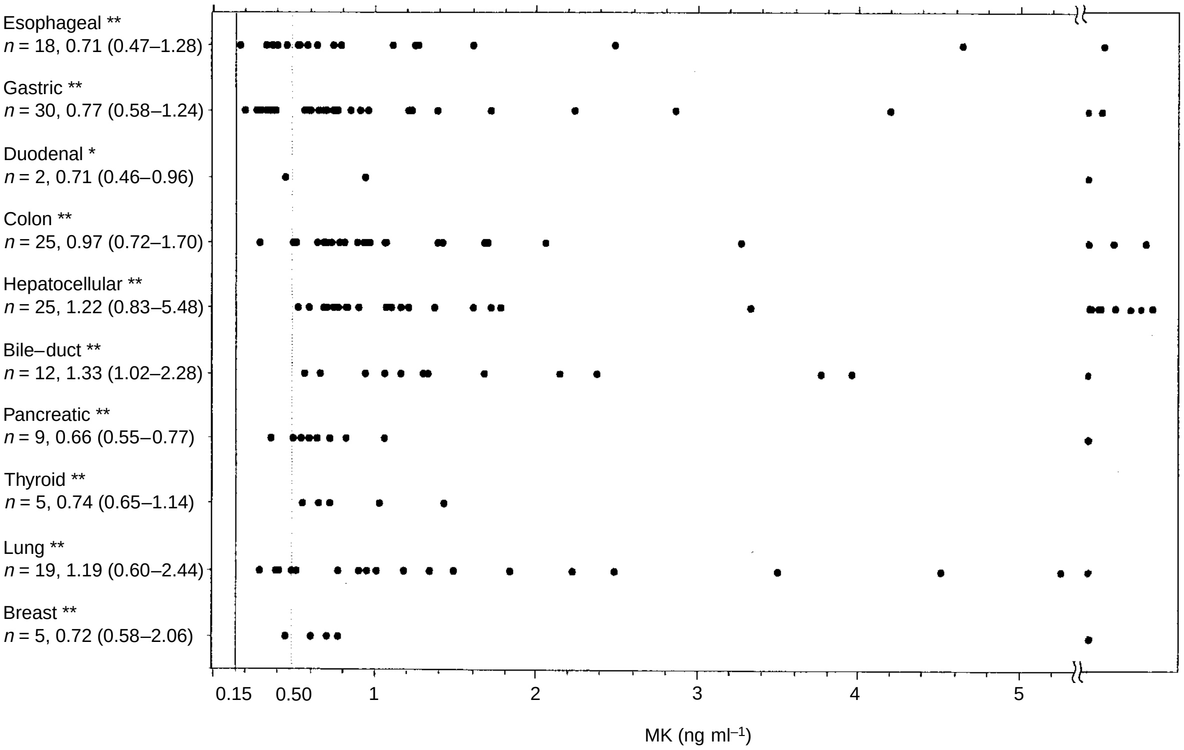

**Figure 3** Comparison of serum MK levels in different types of carcinoma. Solid line at left shows the average value of serum MK in normal individuals. Dotted line shows cut off value of 0.50 ng ml$^{-1}$. Number of cases and the median value with 25th and 75th percentiles are shown under the name of each carcinoma. (Mann-Whitney U-test *$P$ < 0.05; **$P$ < 0.01) Bile-duct carcinoma includes two cases of gallbladder carcinoma women, or among age groups: As an example, in the normal control with ages > 50 ($n$ = 20, average age = 60), the MK value was 0.184 ± 0.111 ng ml$^{-1}$. Serum MK levels of 150 cancer patients showed a profile significantly different from that of normal controls ($P$ < 0.001, Mann–Whitney U test) (Figure 3).

Eighty-seven percent of the patients had a value higher than 0.5 ng ml$^{-1}$.

As shown in Figure 3, the increase in serum MK level was observed in all ten types of carcinomas examined. In addition, each tissue type showed a similar profile of serum MK level, suggesting that the profile of increased serum MK level was general rather than tissue-specific.

In case of gastric carcinomas and lung carcinomas, sera from patients with carcinomas at early stages were available in large numbers to test whether serum MK level was increased at the early stages. We found that statistically significant increase in MK level was observed even at stage I of these carcinomas (Table 2). Although the average value of serum MK was lower in stage I than in stages II–IV, the differences were not statistically significant in both gastric carcinoma and lung carcinoma.

 © 2000 Cancer Research Campaign

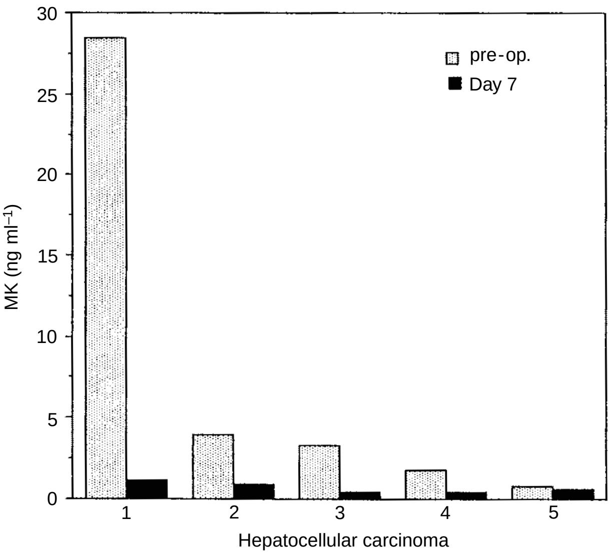

**Figure 4** Effects of tumour removal on serum MK values. MK values were determined before the surgery (▒) or 7 days after the surgery (■). Number indicates the case number

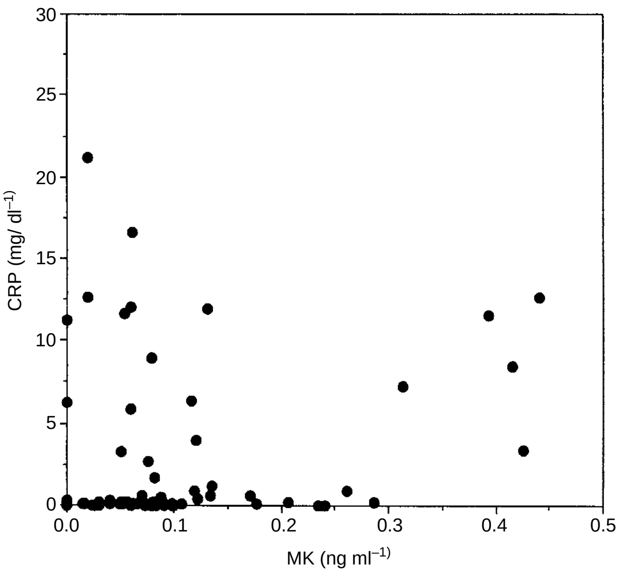

**Figure 5** Relation between C-reactive protein (CRP) and MK. Serum levels of C-reactive protein and MK were examined in 80 patients with or without inflammation. There was no or weak correlation between these two factors

Furthermore, when we compared MK values between patients with different tumour sizes, i.e. > 5 cm diameter, 2–5 cm and < 5 cm, there were no significant differences between the groups.

The effects of surgical removal of tumours on serum MK values were examined in five patients with hepatocellular carcinoma. In four cases, significant decrease of the values was found after tumour removal (Figure 4).

Finally, to determine whether serum MK level was affected by inflammation, we determined serum levels of both CRP and MK of 80 outpatients with inflammation (CRP value > 0.1 mg $dl^{-1}$) or without inflammation. However, as shown in Figure 5, there was no or weak correlation between these two factors ($P$ = 0.0762, correlation coefficient 0.331). Also using the same specimens, we determined leukocyte counts and found no correlation with MK serum levels ($P$ = 0.5697, correlation coefficient – 0.110).

### DISCUSSION

We developed a new procedure for EIA of human MK using rabbit and chicken antibodies. Chicken and mouse MKs show 73% identity, whereas mouse and human MK show 87% identity (Raulais et al, 1991). This sequence divergence between mammals and birds suggests that chickens immunized with human MK could produce antibodies that recognize more epitopes, including epitopes different from those recognized by the rabbit. Indeed, a combination of chicken and rabbit anti-human MK antibodies exhibited a much higher sensitivity in the detection of human MK than rabbit antibodies alone; the major epitopes recognized by chicken antibodies are probably different from those recognized by rabbit antibodies. In EIA for MK reported previously (Muramatsu et al, 1996), we employed rabbit anti-MK antibodies and their biotinylated forms as well as avidin-β-galactosidase and a fluorogenic substrate, 4-methylumbelliferyl-β-D-galactoside. As compared to the previous method, the present EIA was advantageous in that it uses an ordinary colourimetric detection system that is more convenient for clinical use.

Using this procedure, we analysed sera from a large number of patients and found that serum MK levels were elevated in most of the cancer patients examined (87%). The ratio of patients with increased serum MK levels to total cancer patients examined agreed with that of patients with increased MK expression in tumour tissues reported previously (Tsutsui et al, 1993; Garver et al, 1994; Aridome et al, 1995; Nakagawara et al, 1995; O'Brien et al, 1996; Mishima et al, 1997; Song et al, 1997; Ye et al, 1999). Thus, MK expressed in carcinomatous tissues is probably secreted into the bloodstream, leading to an increase in serum MK level in cancer patients. It is notable that the increased expression of MK in carcinomatous tissues and the elevated serum MK levels in cancer patients did not show specification for a particular tissue. This is reminiscent of the case of *p53* mutations in human carcinomas, suggesting the biological importance of MK in carcinogenesis. The role of MK in carcinogenesis and/or tumour progression, as indicated by its transforming (Kadomatsu et al, 1997) and angiogenic (Choudhuri et al, 1997) activities, is consistent with this assumption.

Recently, MK has been shown to have anti-apoptotic activity to embryonic neurons (Michikawa et al, 1993, Owada et al 1999) and to Wilms' tumour cells (Qi et al, 2000). MK also promotes migration of various cells such as embryonic neurons (Maeda et al, 1999), neutrophils (Takada et al, 1997) and macrophages (Horiba et al, 2000). These two activities of MK might be helpful in survival and invasion of tumour cells.

In the majority of cases, the serum MK levels decreased after surgical removal of hepatocellular carcinomas, as is expected from its nature as a tumour marker. Future study is required to know whether the value decreases in all types of tumours. The serum MK values were not significantly dependent on stages and on tumour sizes. The present study also revealed that MK values are not significantly correlated with markers of inflammation, namely CRP values and leukocyte counts. In non-malignant hepatic diseases, 14.5% of patients showed elevated serum MK values (more than 0.5 ng $ml^{-1}$) (Song et al, 1997). Although the frequency

*© 2000 Cancer Research Campaign*

of elevated cases is much less as compared to be patients with carcinomas, it should be kept in mind that in certain restricted cases of non-malignant diseases, serum MK values might become elevated.

We previously found that MK expression was increased in the pre-cancerous tissue at adenoma stages of human colorectal carcinogenesis (Ye et al, 1999). MK was also detected in latent prostate cancers and prostatic intra-epithelial neoplasia (Konishi et al, 1999). These findings imply that MK may be useful in detecting certain carcinomas at early stages. Indeed, in the cases of gastric carcinoma and lung carcinoma, elevated serum MK level was found even at stage I. It is of significant interest whether determination of serum MK could serve as a marker in detection of these carcinomas at the early stages.

### ACKNOWLEDGEMENTS

This work was supported by grant from the Ministry of Education, Science, Sports and Culture, Japan (Grants-in-Aid for COE Research).

### REFERENCES

Aridome K, Tsutsui J, Takao S, Kadomatsu K, Ozawa M, Aikou T and Muramatsu T (1995) Increased midkine gene expression in human gastrointestinal cancers. *Jpn J Cancer Res* **86**: 655–661

Choudhuri R, Zhang HT, Donnini S, Ziche M and Bicknell R (1997) An angiogenic role for the neurokines midkine and pleiotrophin in tumorigenesis. *Cancer Res* **57**: 1814–1819

Garver RI Jr, Radford DM, Donis-Keller H, Wick MR and Milner PG (1994) Midkine and pleiotrophin expression in normal and malignant breast tissue. *Cancer* **74**: 1584–1590

Horiba M, Kadomatsu K, Nakamura E, Muramatsu H, Ikemastu S, Sakuma S, Hayashi K, Yuzawa Y, Matsuo S, Kuzuya M, Kaname T, Hirai M, Saito H and Muramastu T (2000) Neointima formation in a restenosis model is suppressed in midkine-deficient mice. *J Clin Invest* **105**: 489–495

Ishikawa E, Yoshitake S, Imagawa M and Sumiyoshi A (1993) Preparation of monomeric Fab'-horseradish peroxidase conjugate using thiol groups in the hinge and its evaluation in enzyme immunoassay and immunohistochemical staining. *Ann NY Acad Sci* **420**: 74–89

Kadomatsu K, Tomomura M and Muramatsu T (1988) cDNA cloning and sequencing of a new gene intensely expressed in early differentiation stages of embryonal carcinoma cells and in mid-gestation period of mouse embryogenesis. *Biochem Biophys Res Commun* **151**: 1312–1318

Kadomatsu K, Hagihara M, Akhter S, Fan QW, Muramatsu H and Muramatsu T (1997) Midkine induces the transformation of NIH3T3 cells. *Br J Cancer* **75**: 354–359

Kojima S, Muramatsu H, Amanuma H and Muramatsu T (1995) Midkine enhances fibrinolytic activity of bovine endothelial cells. *J Biol Chem* **270**: 9590–9596

Konishi N, Nakamura M, Nakaoka S, Hiasa Y, Cho M, Uemura H, Hirao Y, Muramatsu T and Kadomatsu K (1999) Immunohistochemical analysis of midkine expression in human prostate carcinoma. *Oncology* **57**: 253–257

Kurtz A, Schulte AM and Wellstein A (1995) Pleiotrophin and midkine in normal development and tumour biology. *Crit Rev Oncog* **6**: 151–177

Li YS, Milner PG, Chauhan AK, Watson MA, Hoffman RM, Kodner CM, Milbrandt J and Deuel TF (1990) Cloning and expression of a developmentally regulated protein that induces mitogenic and neurite outgrowth activity. *Science* **250**: 1690–1694

Maeda N, Ichihara-Tanaka K, Kimura T, Kadomatsu K, Muramatsu T and Noda M (1999) A receptor-like protein tyrosine phosphatase (PTP ζ /RPTP β) binds a heparin binding growth factor midkine: Involvement of arginine 78 of midkine in the high affinity binding to PTP ζ. *J Biol Chem* **274**: 12474–12479

Meremies J and Rauvala H (1990) Molecular cloning of the 18-kDa growth-associated protein of developing brain. *J Biol Chem* **265**: 16721–16724

Michikawa M, Kikuchi S, Muramatsu H, Muramatsu T and Kim SU (1993) Retinoic acid responsive gene product, midkine (MK), has neurotrophic functions for mouse spinal cord and dorsal root ganglion neurons in culture. *J Neurosci Res* **35**: 530–539

Mishima K, Asai A, Kadomatsu K, Ino Y, Nomura K, Narita Y, Muramatsu T and Kurino T (1997) Increased expression of midkine during the progression of human astrocytomas. *Neurosci Lett* **233**: 29–32

Muramatsu H, Shirahama H, Yonezawa S, Maruta H and Muramatsu T (1993) Midkine, a retinoic acid-inducible growth/differentiation factor: immunochemical evidence for the function and distribution. *Dev Biol* **159**: 392–402

Muramatsu H, Song XJ, Koide N, Hada H, Tsuji T, Kadomatsu K, Inui T, Kimura T, Sakakibara S and Muramatsu T (1996) Enzyme-linked immunoassay for midkine, and its application to evaluation of midkine levels in developing mouse brain and sera from patients with hepatocellular carcinomas. *J Biochem* **119**: 1171–1175

Nakagawara A, Milbrandt J, Muramatsu T, Deuel TF, Zhao H, Cnaan A and Brodeur GM (1995) Differential expression of pleiotrophin and midkine in advanced neuroblastomas. *Cancer Res* **55**: 1792–1797

Owada K, Saijo N, Kobayashi T, Mizusawa H, Muramatsu H, Muramatsu T and Michikawa, M (1999) Midkine inhibits caspase-dependent apoptosis via the activation of mitogen-activated protein kinase and phosphatidyl-inositol 3-kinase in cultured neurons. *J Neurochem* **73**: 2084–2092

O'Brien T, Cranston D, Fuggle S, Bicknell R and Harris AL (1996) The angiogenic factor midkine is expressed in bladder cancer, and overexpression correlates with a poor outcome in patients with invasive cancers. *Cancer Res* **56**: 2515–2518

Qi M, Ikematsu S, Ichihara-Tanaka K, Sakuma S, Muramatsu T and Kadomatsu K (2000) Midkine rescues Wilms' tumour cells from cisplatin-induced apoptpsis: regulation of Bcl-2 expression by midkine. *J Biochem* **127**: 269–277

Raulais D, Lagente-Chevallier O, Guettet C, Duprez D, Courtois Y and Vigny M (1991) A new heparin-binding protein regulated by retinoic acid from chick embryos. *Biochem Biophys Res Commun* **174**: 708–715

Song XJ, Muramatsu H, Aridome K, Aikou T, Koide N, Tsuji T and Muramatsu T (1997) The serum level of midkine, a heparin-binding growth factor, as a tumour marker. *Biomed Res* **18**: 375–381

Takada T, Toriyama K, Muramatsu H, Song XJ, Torii S and Muramatsu T (1997) Midkine, a retinoic acid-inducible heparin-binding cytokine in inflammatory responses: chemotactic activity to neutrophils and association with inflammatory synovitis. *J Biochem* **122**: 453–458

Tsutsui J, Kadomatsu K, Matsubara S, Nakagawara A, Hamanoue M, Takao S, Shimazu H, Ohi Y and Muramatsu T (1993) A new family of heparin-binding growth/differentiation factors: increased midkine expression in Wilms' tumour and other human carcinomas. *Cancer Res* **53**: 1281–1285

Ye C, Qi M, Fan QW, Ito K, Akiyama S, Kasai Y, Matsuyama M, Muramatsu T and Kadomatsu K (1999) Expression of midkine in the early stage of carcinogenesis in human colorectal cancer. *Br J Cancer* **79**: 179–184

 *© 2000 Cancer Research Campaign*
